# Boosting the discriminatory power of sparse survival models via optimization of the concordance index and stability selection

**DOI:** 10.1186/s12859-016-1149-8

**Published:** 2016-07-22

**Authors:** Andreas Mayr, Benjamin Hofner, Matthias Schmid

**Affiliations:** Institut für Medizininformatik, Biometrie und Epidemiologie, Friedrich-Alexander-Universität Erlangen-Nürnberg (FAU), Waldstr. 6, Erlangen, 91054 Germany; Institut für Medizinische Biometrie, Informatik und Epidemiologie, Rheinische Friedrich-Wilhelms-Universität Bonn, Sigmund-Freud-Str. 25, Bonn, 53105 Germany

**Keywords:** Time-to-event data, Boosting, Stability selection, Concordance index, Variable selection, High-dimensional data

## Abstract

**Background:**

When constructing new biomarker or gene signature scores for time-to-event outcomes, the underlying aims are to develop a discrimination model that helps to predict whether patients have a poor or good prognosis and to identify the most influential variables for this task. In practice, this is often done fitting Cox models. Those are, however, not necessarily optimal with respect to the resulting discriminatory power and are based on restrictive assumptions. We present a combined approach to automatically select and fit sparse discrimination models for potentially high-dimensional survival data based on boosting a smooth version of the concordance index (*C*-index). Due to this objective function, the resulting prediction models are optimal with respect to their ability to discriminate between patients with longer and shorter survival times. The gradient boosting algorithm is combined with the stability selection approach to enhance and control its variable selection properties.

**Results:**

The resulting algorithm fits prediction models based on the rankings of the survival times and automatically selects only the most stable predictors. The performance of the approach, which works best for small numbers of informative predictors, is demonstrated in a large scale simulation study: *C*-index boosting in combination with stability selection is able to identify a small subset of informative predictors from a much larger set of non-informative ones while controlling the per-family error rate. In an application to discover biomarkers for breast cancer patients based on gene expression data, stability selection yielded sparser models and the resulting discriminatory power was higher than with lasso penalized Cox regression models.

**Conclusion:**

The combination of stability selection and *C*-index boosting can be used to select small numbers of informative biomarkers and to derive new prediction rules that are optimal with respect to their discriminatory power. Stability selection controls the per-family error rate which makes the new approach also appealing from an inferential point of view, as it provides an alternative to classical hypothesis tests for single predictor effects. Due to the shrinkage and variable selection properties of statistical boosting algorithms, the latter tests are typically unfeasible for prediction models fitted by boosting.

**Electronic supplementary material:**

The online version of this article (doi:10.1186/s12859-016-1149-8) contains supplementary material, which is available to authorized users.

## Background

In the evaluation of biomarkers and gene signatures for survival data, the focus is often on the ability of a new marker combination to discriminate between patients with larger and smaller survival times [1–3]. For example, one is often interested in predicting whether patients survive a specific time point of interest, e.g., five years after baseline examination. In practice, prediction models are often derived using Cox regression, which, however, suffers from restrictive regularity assumptions such as the proportional hazards assumption. It is well known that, if violated, these assumptions may cause Cox regression to result in suboptimal model fits with a decreased prediction accuracy [4].

Despite its limitations, Cox regression remains the predominant technique for modeling survival data in biostatistics (see [3, 5] for recent examples). In fact, most attempts to relax the proportional hazards assumption (such as stratification of the baseline hazard and time-varying coefficients) retain the basic properties and limitations of the Cox model. Similarly, regularization schemes for survival models (such as penalized Cox regression [6, 7], and univariate preselection of markers [8]) are usually based on Cox modeling. On the other hand, non-Cox-based approaches from the machine learning field such as support vector machines for survival data [9] or random survival forests [10] have the problem that they lead to black box predictions with limited interpretability.

In this paper we focus on a statistical modelling approach that results in additive predictors

$$ \eta := \beta_{0} + \sum_{l =1}^{p} \beta_{l} \cdot x_{l} = X^{\top}{\boldsymbol \beta} $$

which are optimized with respect to the *concordance index for survival data* (often denoted as Harrell’s *C* or *C*-index) [11–13]. The *C*-index is a discrimination measure for the evaluation of prediction models. The *C*-index is not based on restrictive regularity assumptions (in contrast to Cox regression) but is non-parametric in the sense that it only evaluates to which extent the *ranking* of the values of the linear combination *η* is in agreement with the ranking of the survival times. In a recent article [14] it was shown that the *C*-index can be optimized efficiently via a gradient boosting approach (“*C*-index boosting”), which is also feasible in high-dimensional data situations. Since the *C*-index is a popular evaluation criterion in bioinformatics and biostatistics [15–17], the method proposed in [14] has the additional advantage that optimization of the *C*-index results in prediction rules that focus directly on the performance measure of interest instead of using a different optimization criterion such as the partial log-likelihood in Cox regression.

Despite its good performance, especially in situations where the proportional hazards assumption is violated, *C*-index boosting has the drawback that variable selection cannot be accomplished as easily as with traditional boosting algorithms designed for the *calibration* of a prediction model (such as LogitBoost [18, 19], which optimizes the conditional probability estimates for a binary outcome). In fact, the *discriminatory* nature of the *C*-index, which evaluates the ranking of the values of *η* but does not involve probability estimation, has been observed to be relatively insensitive to overfitting, making traditional regularization approaches for boosting (such as early stopping [20]) infeasible. This observation coincides with a recent result by Wyner et al. [21] who demonstrated that overfitting in boosting models for binary outcomes is unlikely to happen as long as discriminatory measures (such as the percentage of observations correctly classified) are used for evaluation. While resistance to overfitting is often considered to be an advantage in machine learning research, it also implies that sparse prediction rules, which are desirable in biomedical applications for reasons of interpretability and generalizability [22], are difficult to obtain.

To address this problem, we propose a new variable selection technique for *C*-index boosting that is able to identify the most influential and stable predictors for survival. The method is based on the stability selection approach proposed by Meinshausen and Bühlmann [23], which has recently been enhanced [24] and adapted to gradient boosting estimation [25, 26]. The idea of stability selection is to fit the model to a high number of subsets of the original data. One then determines the average number of subsets in which a variable was selected. Variables where the selection frequency exceeds a certain threshold are considered to be stable. Importantly, variable selection is accomplished via controlling the per-family error rate (PFER) of the predictor variables selected for inclusion in the boosting model. As a consequence, the sparsity of the resulting prediction model is governed by the desired level of error control, and resistance to overfitting is no longer an issue. Using a comprehensive simulation study, as well as a gene expression data set on lymph node negative breast cancer collected by Desmedt et al. [27], we will demonstrate that stability selection can also be adapted to perform variable selection in *C*-index boosting. In particular, our results suggest that the new method is able to both optimize the *C*-index and to identify the most relevant predictors for survival at the desired error level.

## Methods

### The *C*-index for survival data

The concordance index evaluates the rank-based concordance probability between a continuous predictor *η* and the outcome [11, 12]. The non-parametric criterion can be applied for continuous, ordinal and dichotomous outcomes, as well as for time-to-event outcomes. In the latter case, it is defined as

$$C := \mathbb{P}(\eta_{j} > \eta_{i} \, | \, T_{j} < T_{i}) \,, $$

where *T*_*j*_, *T*_*i*_ are the survival times and *η*_*j*_ and *η*_*i*_ the predictors of two observations in an i.i.d. test sample. The *C*-index measures whether large values of *η* are associated with short survival times *T* and vice versa. The interpretation is similar to the widely known AUC (area under the receiver operating characteristics curve): A *C*-index of 1 represents a perfect discrimination while a *C*-index of 0.5 will be achieved by a completely non-informative marker. In fact, it was shown that the *C*-index is equivalent to the area under the time-dependent receiver operating characteristics (ROC) curve, which summarizes the discriminatory power of *η* over all time points [1].

An extension of the *C*-index that evaluates the concordance probability between *η* and *T* up to a pre-specified time point *τ* is the *truncatedC*-index

$$C_{\text{tr}} := \mathbb{P}(\eta_{j} > \eta_{i} \, | \, T_{j} < T_{i}, \, T_{j} \leq \tau) \,. $$

The truncated *C*-index is an alternative to the *C*-index in situations where the right tail of the estimated survival function of *T* is unstable [1, 28–30]. While we do not explicitly consider the truncated version of the *C*-index in this work, our methodology easily extends to truncated time ranges of the form [0,*τ*] (see below).

Although following a relatively simple and straight-forward definition, in practice the estimation of the *C*-index becomes problematic in samples with censoring. Some estimators proposed in the literature omit observation pairs where the smaller survival time was censored, however this can lead to biased results [31]. Others rely on the assumptions of a Cox proportional hazards model [32, 33] which becomes problematic in settings were those are not fulfilled. For an overview and comparison of different estimators for the *C*-index and other discriminatory measures for survival data see Schmid and Potapov [13].

To overcome these issues, Uno et al. [28] proposed an asymptotically unbiased estimator which incorporates inverse probability of censoring weighting [34]:

$$ \widehat{C}_{\text{Uno}}(T, \eta) \ := \ \frac{\sum_{j,i} \frac{\Delta_{j}}{ \hat{G}(\tilde{T}_{j})^{2}} \, \mathrm{I} (\tilde{T}_{j} < \tilde{T}_{i}) \mathrm{I} \left(\hat{\eta}_{j} > \hat{\eta}_{i} \right) }{\sum_{j,i} \frac{\Delta_{j}}{ \hat{G}(\tilde{T}_{j})^{2}} \, \mathrm{I} (\tilde{T}_{j} < \tilde{T}_{i}) }. $$

The term $\frac {\Delta _{j}}{\hat {G}(\tilde {T}_{j})^{2}}$ accounts for the inverse probability that observation *j* is censored. *Δ*_*j*_ represents the censoring indicator, $\tilde {T}$ are observed survival times subject to censoring and $\hat {G}(\cdot)$ denotes the Kaplan-Meier estimator of the unconditional survival function for the censoring time *T*_cens_ (estimated from the learning data via the observed $\tilde {T}$ and taking *Δ*_*j*_ as event indicator).

When a truncated time range [0,*τ*] is considered, the truncated *C*-index can be estimated by an extension of $\widehat {C}_{\text {Uno}}(T, \eta)$ defined by (c.f., [30])

$$\begin{array}{@{}rcl@{}} \widehat{C}_{\text{tr}}(T, \eta, \tau) := \frac{\sum_{j,i} \Delta_{j} \Delta_{i} \mathrm{I} (\tilde{T}_{j} < \tilde{T}_{i}, \tilde{T}_{j} \leq \tau) \mathrm{I} \left(\hat{\eta}_{j} > \hat{\eta}_{i} \right) }{\sum_{j,i} \Delta_{j} \Delta_{i} \mathrm{I} (\tilde{T}_{j} < \tilde{T}_{i}, \tilde{T}_{j} \leq \tau) } \end{array} $$

Of note, the estimator $\widehat {C}_{\text {Uno}}(T, \eta)$ is a consistent estimator of the *C*-index if censoring is independent of *T* (*coarsening completely at random*, [28, 30]). If censoring is independent of *T* conditional on *η* (*coarsening at random*), the terms $\hat {G}(\cdot)$ in the definition of $\widehat {C}_{\text {Uno}}(T, \eta)$ can be replaced by conditional terms $\hat {G}(\cdot | \eta)$ that are derived from a survival model for the censoring distribution [29, 30]. Wang and Long (2016) also analyzed the properties of $\widehat {C}_{\text {Uno}}(T, \eta)$ in situations where censoring is not independent of *T*.

### Boosting the *C*-index

To find the optimal predictor *η* with respect to the *C*-index, we adapt a component-wise gradient boosting algorithm [35] with simple linear models as base-learners. Boosting originally emerged from machine learning, but during the last 15 years has evolved into a powerful tool to fit statistical models (“statistical boosting”, [36, 37]). The basic idea is to apply simple regression functions as base-learners (in the easiest case simple linear models) and iteratively fit them one-by-one to the negative gradient of a loss function. In every boosting iteration only the best-fitting base-learner is included in the model, effectively leading to variable selection.

The loss function defines the type of regression setting the additive predictor is optimized for. The *L*_2_ squared error loss leads to classical regression of the mean [38], the *L*_1_ loss to median regression which can be extended to quantile regression via the check-function [39]. Incorporating the negative log-likelihood as loss function allows to fit classical generalized linear or additive models (GLMs or GAMs, [35]). For an overview of different loss functions for gradient boosting and their implementation see Hofner et al. [40].

Using Uno’s estimator for the *C*-index as loss function, however, is unfeasible because $\widehat {C}_{\text {Uno}}(T, \eta)$ is not differentiable with respect to *η*. To solve this problem, we approximate the indicator function $\mathrm {I}(\hat {\eta }_{j} > \hat {\eta }_{i})$ by a sigmoid function (similar to Ma and Huang [41])

$$K(\hat{\eta}_{j} - \hat{\eta}_{i}) = 1 / \left(1 + \exp \left(-\frac{(\hat{\eta}_{j} - \hat{\eta}_{i}) }{ \sigma}\right)\right), $$

leading to a smooth estimator of $\widehat {C}_{\text {Uno}}$

$$ \widehat{C}_{\text{smooth}}(T, \hat{\eta}) \ = \ \ \frac{\sum_{j,i} \frac{\Delta_{j}}{\hat{G}(\tilde{T}_{j})^{2}} \, \mathrm{I} (\tilde{T}_{j} < \tilde{T}_{i}) \cdot K (\hat{\eta}_{j} - \hat{\eta}_{i}) } { \sum_{j,i} \frac{\Delta_{j}}{\hat{G}(\tilde{T}_{j})^{2}} \, \mathrm{I} (\tilde{T}_{j} < \tilde{T}_{i}) } $$

which is differentiable with respect to *η* and will serve as loss function for the algorithm. A more detailed overview on the algorithm for boosting the *C*-index and its application is provided in the Additional file 1.

The variable selection properties of statistical boosting algorithms are controlled by the stopping iteration *m*_stop_ [20]. If the algorithm is stopped before convergence (*early stopping*), variables that have never been selected up to this iteration are effectively excluded from the final model. The stopping iteration *m*_stop_ is typically chosen such that it optimizes the prediction accuracy on separate test data generated via resampling techniques (e.g., bootstrapping or subsampling).

In case of *C*-index boosting, this common procedure, however, becomes problematic as the rank-based loss function is rather robust against overfitting and early stopping is hardly possible. An optimal *m*_stop_ often cannot be determined in this case. Similar results have been described for binary outcomes if discriminatory measures are used to evaluate the prediction performance [21]. In case of *C*-index boosting, in many practical settings it hence makes sense to run the algorithm until convergence and omit the optimization of *m*_stop_ (see [14]).

### Stability selection

To ensure the selection of the most influential predictors despite this resistance to overfitting, we incorporate the stability selection approach by Meinshausen and Bühlmann [23] which was later refined by Shah and Samworth [24]. Stability selection is a generic method that applies to a wide range of statistical estimation techniques which conduct variable selection [42], including penalized regression approaches such as lasso [43], boosting [18] or tree based approaches such as random forests [44].

The principle idea is to use subsamples of size n/2 and fit a boosting model on each of the *B* subsamples until a pre-specified number of variables *q* out of the *p* possible predictor variables is selected. Average selection probabilities $\hat {\pi }_{j}$ are computed for each predictor (*j*=1,…,*p*) and only variables that exceed a pre-specified threshold *π*_thr_ are included in the final model. An important advantage of stability selection is that it controls the per-family error rate $\text {PFER} = \mathbb {E}(V)$, where *V* is the number of false positive variables, and thus provides error bounds for the number of false positives. An upper bound to the PFER (depending on *p*, *q* and *π*_thr_) can be derived as

$$ \mathbb{E}(V) \leq \frac{q^{2}}{(2\pi_{\text{thr}} - 1) p} $$

under certain conditions [23].

Shah and Samworth [24] propose to use 2·*B* complementary pairs, i.e., use the subsample as well as its complement. With additional assumptions on the distribution of the selection frequencies (unimodality or *r*-concavity), tighter error bounds can be derived [24]. This *r*-concavity can be seen as an interpolant between unimodality and log-concavity. With *r*=−*∞r*-concavity equals the unimodality assumption and with *r*=0 log-concavity is assumed (for a thorough definition see [24]). Error bounds with unimodality assumption are tighter than the standard error bounds from the equation above, but not as tight as error bounds with r-concavity assumption. Usually, both assumptions hold [24].

The selection of the parameters *q*, *π*_thr_ and PFER are crucial for the performance of stability selection. In general, we advice to choose *q* large enough to select all influential variables but small enough to reflect the researchers believe in the sparsity of the resulting model. In a sensible range, the actual size of *q* is of minor importance. Similarly, Meinshausen and Bühlmann [23] found that the actual choice of the threshold *π*_thr_ is of minor importance as long as it is in a sensible range (∈(0.6,0.9)). Note that for a fixed *q* it is computationally very easy to change either the threshold or the PFER as the resampling results can be reused. Hence, for fixed *q* different thresholds (corresponding to different levels of error control) can be easily compared. Larger thresholds lead to sparser models, while thresholds close to 0.5 lead to models which are less sparse. This is also reflected in the upper bound for the PFER which decreases with increasing threshold. Selection frequencies resulting from stability selection can also be used as a descriptive statistic to assess which variables are selected with high frequencies and which variables are rarely selected.

If error control is of primary interest, we advice to chose *q* and the upper bound for the PFER. The PFER should be chosen such that *α*≤PFER≤*m*·*α*, with significance level *α* and *m* hypothesis tests. This provides a good rationale for a sensible error control with the extreme cases of FWER-control (family-wise error rate; PFER=*α*) and no multiplicity adjustment (PFER=*m*·*α*).

For an in-depth overview of stability selection in the context of boosting, see Hofner et al. [26].

### Implementation

All presented methods are made available for the open source statistical programming environment R [45]. The algorithm for boosting the *C*-index is implemented via the Cindex() family for the add-on package mboost. Stability selection is implemented via the stabsel() function from the stabs [46] package, which is also incorporated in mboost. It provides an implementation of the classical approach [23] and the extended sampling scheme using complementary pairs [24]. For evaluating the discriminatory power of the resulting models on test data, Uno’s estimator for the *C*-index the is provided with the UnoC() function of the survAUC [47] package. A worked-out example on how to apply these function in practice is provided in Additional file 1, the R-code to reproduce the analyses of this article is included as Additional file 2. In order to benchmark our results, we used the competing Cox lasso approach implemented in the glmnet package [48] which also can be combined with stability selection via stabs. Note that also other implementations for boosting survival models are available in the R framework (gbm [49], CoxBoost [50]) as well as methods depending on the Brier score [51], like the peperr [52] and the pec [53] packages.

## Results

### Simulation study

We carried out a simulation study to check the performance of stability selection in combination with *C*-index boosting under known conditions. The aims of the simulation study were:

- To analyze if the algorithm is able to correctly identify a small subset *p*_inf_ of informative variables from a larger set of *p* possible predictors in settings of *p*>*n*.
- To investigate the impact of the two parameters which have to be specified for stability selection, namely the number of selected variables *q* per boosting run and the threshold *π*_thr_ for the necessary selection probability.
- To compare the resulting discriminatory power of the final models (containing only *stable* predictors) with the ones from *C*-index boosting without stability selection and the competing Cox lasso approach.
- To check the performance of our approach in scenarios where the proportional hazards assumption does not hold.

The survival times *T* were simulated from a log-logistic distribution for accelerated failure time (AFT) models [54] and are based on the model equation

$$ \log(T) = \mu + \phi \cdot W \, $$

where *μ* and *ϕ* are location and scale parameters, and *W* is a noise variable following a standard logistic distribution. The *true* underlying model was *μ*=*x*^⊤^*β* with *β*=(1.5,1,−1,−1.5,0,...,0)^⊤^ for *p*_inf_=4 and was correspondingly extended for other numbers of informative predictors *p*_inf_∈{4,12,40}. The predictors *X*_1_,....,*X*_*p*_ were drawn from a multivariate normal distribution with pairwise correlation (*ρ*=0.5) and *p*∈{50,500,1000}. Note that only a very small amount *p*_inf_ of the *p* available predictors have an actual effect on the survival time. In scenarios where the proportional hazard assumption should not be fulfilled, also the scale parameter *ϕ* depended on a predictor variable *ϕ*= exp(*x*_1_)/5, otherwise it was a simple scalar (c.f., [13]).

Additionally to the survival times *T*, we generated for every observation *i*=1,...,*n* an additional censoring time *T*_cens*i*_ and defined the observed survival time by $\tilde {T}_{i} := \min (T_{i}, T_{\text {cens}i})$ leading to independent censoring of on average 50 % of the observations. The sample size remained fixed with *n*=200 observations. For stability selection we used 2·*B*=100 complementary subsamples and computed the error bounds under the r-concavity assumption (cf., [26]). The final models containing only the selected stable variables were fitted with a fixed *m*_stop_=1000. We compared the performance of this approach also with *C*-index boosting on all *p* predictors (also without tuning, but with fixed *m*_stop_=10000) and the Cox lasso. For the latter, the shrinkage parameter was optimized via 10-fold cross-validation.

#### Variable selection

First, we compared the selection rates for different values of *q* and *π*_thr_. The median number of true and false positives from 100 simulation runs for the different scenarios are presented in Table 1. One can observe that the algorithm is able to correctly identify the true informative predictors out of up to 1000 possible predictors in case of *p*_inf_=4: In all combinations of *q* and *π*_thr_ the four true informative variables were included in the final model if at least four variables had been selected at all. The latter especially becomes a problem if *q* was chosen too small with respect to *p* (e.g., *q*=5 for *p*=1000). These results also hold if the proportional hazard assumption is violated.

**Table 1:** Variable selection results from 100 simulation runs: median number of true positives | false positives and calculated upper bound for the per-family-error rate (PFER, in brackets) for different values of *q* and *π*_thr_

					*C*-index boosting						Cox
*p*	*p*_inf_	*n*	PH-viol	*q*	*π*_thr_ = 0.5	*π*_thr_ = 0.6	*π*_thr_ = 0.7	*π*_thr_ = 0.8	*π*_thr_ = 0.9	without *π*_thr_	lasso
1000	4	200	false	100	4 |8 (24.8)	4 |3 (11.4)	4 |1 (4.27)	4 |0 (1.92)	4 |0 (0.75)	4 |180	4 |36
				50	4 |1 (5.20)	4 |0 (2.61)	4 |0 (0.97)	4 |0 (0.43)	4 |0 (0.17)		
				20	4 |0 (0.61)	4 |0 (0.33)	3 |0 (0.14)	3 |0 (0.06)	2 |0 (0.02)		
				15	4 |0 (0.32)	3 |0 (0.17)	3 |0 (0.08)	3 |0 (0.04)	2 |0 (0.01)		
				10	3 |0 (0.13)	3 |0 (0.07)	3 |0 (0.04)	2 |0 (0.02)	2 |0 (0.01)		
				5	2 |0 (0.03)	2 |0 (0.02)	2 |0 (0.01)	2 |0 (0.00)	1 |0 (0.00)		
500	4	200	false	100	4 |14 (51.9)	4 |5 (27.9)	4 |2 (10.4)	4 |0 (4.73)	4 |0 (1.87)	4 |166	4 |31
				50	4 |3 (12.4)	4 |1 (5.71)	4 |0 (2.13)	4 |0 (0.96)	4 |0 (0.38)		
				20	4 |0 (1.55)	4 |0 (0.82)	4 |0 (0.30)	3 |0 (0.14)	3 |0 (0.05)		
				15	4 |0 (0.79)	4 |0 (0.44)	3 |0 (0.17)	3 |0 (0.07)	3 |0 (0.03)		
				10	4 |0 (0.31)	3 |0 (0.16)	3 |0 (0.07)	3 |0 (0.03)	2 |0 (0.01)		
				5	3 |0 (0.07)	3 |0 (0.03)	2 |0 (0.02)	2 |0 (0.01)	1 |0 (0.00)		
500	4	200	true	100	4|13 (51.9)	4|5 (27.9)	4|2 (10.4)	4|0 (4.73)	4|0 (1.87)	4 |171	4 |36
				50	4|2 (12.4)	4|1 (5.71)	4|0 (2.13)	4|0 (0.96)	4|0 (0.38)		
				20	4|0 (1.55)	4|0 (0.82)	4|0 (0.30)	4|0 (0.14)	3|0 (0.05)		
				15	4|0 (0.79)	4|0 (0.44)	4|0 (0.17)	3|0 (0.07)	3|0 (0.03)		
				10	4|0 (0.31)	4|0 (0.16)	3|0 (0.07)	3|0 (0.03)	2|0 (0.01)		
				5	3|0 (0.07)	3|0 (0.03)	2|0 (0.02)	2|0 (0.01)	1|0 (0.00)		
50	4	200	false	20	4 |7 (50.0)	4 |4 (50.0)	4 |2 (6.33)	4 |1 (3.06)	4 |0 (1.25)	4 |43	4 |14
				15	4 |3 (50.0)	4 |2 (8.12)	4 |1 (2.88)	4 |0 (1.34)	4 |0 (0.54)		
				10	4 |1 (5.19)	4 |0 (2.79)	4 |0 (1.04)	4 |0 (0.47)	4 |0 (0.19)		
				5	4 |0 (1.24)	4 |0 (0.57)	4 |0 (0.21)	4 |0 (0.10)	3 |0 (0.04)		
500	12	200	false	100	12|12 (51.9)	12|4 (27.9)	12|1 (10.4)	11|0 (4.73)	9|0 (1.87)	12 |150	12 |78
				50	9|2 (12.4)	8|0 (5.71)	7|0 (2.13)	6|0 (0.96)	3|0 (0.38)		
				20	5|0 (1.55)	4|0 (0.82)	3|0 (0.30)	2|0 (0.14)	1|0 (0.05)		
				15	4|0 (0.79)	3|0 (0.44)	2|0 (0.17)	1|0 (0.07)	0|0 (0.03)		
				10	3|0 (0.31)	2|0 (0.16)	1|0 (0.07)	0|0 (0.03)	0|0 (0.01)		
500	40	200	false	200	17|13 (500)	12|5 (500)	8|2 (63.3)	4|0 (30.6)	1|0 (12.5)	35 |139	9 |12
				100	16|12 (51.9)	11|4 (27.9)	7|2 (10.4)	4|0 (4.73)	1|0 (1.87)		
				50	6|2 (12.4)	4|1 (5.71)	2|0 (2.13)	1|0 (0.96)	0|0 (0.38)		
				25	2|0 (2.6)	1|0 (1.3)	0|0 (0.48)	0|0 (0.21)	0|0 (0.08)		

In every setting *p*_inf_ predictors were truly informative, *p*−*p*_inf_ were non-informative; PH-viol: settings were the proportional hazards assumption was violated. *C*-index boosting without stability selection (without *π*_thr_) was fitted on all *p* predictors with a fixed large *m*_stop_; in case of the Cox lasso the shrinkage parameter was optimized via 10-fold cross-validation

For given *q*, the parameter *π*_thr_ controls the sparsity of the resulting models: For *p*=1000, *q*=100 and *p*_inf_=4, for example, on average eight variables were falsely selected with a threshold value of *π*_thr_=0.5. This number decreased over three (*π*_thr_=0.6), and one (*π*_thr_=0.7) to zero for higher threshold values *π*_thr_. Thus, for threshold values of *π*_thr_≥0.8 only the four informative predictors were included in the final model.

Comparing the results for *p*_inf_=4 and different numbers of predictors *p*, it gets clear that the optimal combination of *q* and *π*_thr_ depends not only on the number of true informative variables but also on *p*. For larger numbers of *p*, *q* should also be larger to give the algorithm the chance to select enough variables on each subsample so that the informative ones pass the threshold: For *p*=50 this could be achieved already with *q*=5; for *p*=1000 at least *q*=15 is necessary (better results for *q*=50 or higher). This interdependence between *q*, *p* and *π*_thr_ can be also observed via the computed upper bound for the PFER (following the error bounds provided in [24]). It has to be noted, however, that on average much less variables were falsely selected in practice than could be in theory (following the upper bound of the PFER). This indicates that the error bound is conservatively controlled.

For higher numbers of informative variables *p*_inf_ the algorithm had more problems identifying the correct ones. For *p*_inf_=12, the number of selected variables per subsample has to be increased to *q*=100 to incorporate all true informative ones. For smaller values of *q*, even for *π*_thr_=0.5 only parts of the true predictors were selected; however, stability selection still mostly prevented incorporating false positives. The competing Cox lasso approach, in contrast, also on average achieved to identify the true *p*_inf_=12, but additionally included large numbers of non-informative variables in the final model. For *p*_inf_=40, the picture became more extreme: Now both approaches, *C*-index boosting with stability selection and the Cox lasso were no longer able to select the correct predictors. Only for *q*=200 and *π*_thr_=0.5 on average 17 out of 40 predictors were correctly identified by *C*-index boosting with stability selection (13 false positives), the Cox lasso incorporated 9 true predictors in the model (12 false positives). *C*-index boosting without stability selection in this case correctly identified 35 predictors.

#### Discriminatory power

The discriminatory power of the final models was evaluated on independent test data with *n*=1000 observations. The resulting median *C*-index values (obtained from Uno’s original estimator) for the different scenarios are presented in Table 2. The estimates for $\hat {C}_{\text {Uno}}$ reflect the results from the variable selection in Table 1: The highest discriminatory power was achieved if the correct variables had been identified as stable predictors and were included in the final model. For truly sparse models (*p*_inf_=4), this could be either achieved via large values of *q* and high thresholds (e.g., *q*=100 and *π*_thr_=0.9 for *p*=1000) or smaller values of *q* and therefore also lower thresholds (e.g., *q*=15 and *π*_thr_=0.5 for *p*=500). For larger true models (*p*_inf_=12), a high discriminatory power could only be achieved when enough variables were included: Best results were found for combinations with large *q* and small thresholds ($\hat {C}_{\text {Uno}} = 0.9218$ for *q*=100 and *π*_thr_=0.6). The poorest discriminatory power from our approach resulted from the scenarios with *p*_inf_=40 ($\hat {C}_{\text {Uno}} = 0.6416$ for *q*=200 and *π*_thr_=0.5). In this case, with a rather large number of informative predictors, the additional stability selection even led for all combinations of *q* and *π*_thr_ to poorer results than standard boosting of the *C*-index (cf., results with *p*_inf_=50 of Meinshausen and Bühlmann [23]).

**Table 2:** Resulting discriminatory power of *C*-index boosting in combination with stability selection for different values of *q* and *π*_thr_ compared to the competing Cox lasso approach

					*C*-index boosting						Cox
*p*	*p*_inf_	*n*	PH-viol	*q*	*π*_thr_ = 0.5	*π*_thr_ = 0.6	*π*_thr_ = 0.7	*π*_thr_ = 0.8	*π*_thr_ = 0.9	without *π*_thr_	lasso
1000	4	200	false	100	0.8150	0.8286	0.8358	0.8393	0.8396	0.7889	0.8148
				50	0.8343	0.8365	0.8381	0.8357	0.8253		
				20	0.8324	0.8252	0.7829	0.7662	0.7394		
				15	0.8309	0.7813	0.7694	0.7519	0.7340		
				10	0.7799	0.7683	0.7519	0.7426	0.6202		
				5	0.7497	0.7426	0.7323	0.6176	0.5993		
500	4	200	false	100	0.7998	0.8179	0.8305	0.8361	0.8391	0.7735	0.8161
				50	0.8268	0.8332	0.8375	0.8388	0.8340		
				20	0.8358	0.8351	0.8309	0.7744	0.7607		
				15	0.8346	0.8314	0.7835	0.7672	0.7521		
				10	0.8279	0.7801	0.7672	0.7587	0.7400		
				5	0.7627	0.7587	0.7444	0.7347	0.6154		
500	4	200	true	100	0.8304	0.8481	0.8612	0.8656	0.8671	0.7886	0.8345
				50	0.8555	0.8635	0.8664	0.8668	0.8664		
				20	0.8657	0.8654	0.8626	0.8477	0.7662		
				15	0.8654	0.8626	0.8554	0.7743	0.7573		
				10	0.8598	0.8442	0.7757	0.7614	0.7360		
				5	0.7660	0.7573	0.7391	0.7275	0.6219		
50	4	200	false	20	0.8183	0.8248	0.8303	0.8333	0.8358	0.7939	0.8256
				15	0.8268	0.8298	0.8329	0.8353	0.8370		
				10	0.8314	0.8348	0.8366	0.8370	0.8366		
				5	0.8373	0.8353	0.8324	0.8247	0.7662		
500	12	200	false	100	0.9109	0.9218	0.8996	0.8639	0.8081	0.8852	0.8834
				50	0.7991	0.7880	0.7451	0.7089	0.6482		
				20	0.6954	0.6609	0.6239	0.5698	–		
				15	0.6664	0.6274	0.5830	0.5549	–		
				10	0.6275	0.5848	0.5610	–	–		
500	40	200	false	200	0.6416	0.6269	0.6088	0.5755	0.5344	0.6983	0.5782
				100	0.6373	0.6245	0.6028	0.5706	0.5308		
				50	0.5907	0.5703	0.5407	0.5129	–		
				25	0.5411	0.5269	–	–	–		

In case of *C*-index boosting, the final models were fitted with fixed *m*_stop_=1000. Numbers represent the median $\hat {C}_{\text {Uno}}$ on test samples from 100 simulation runs. PH-viol: settings were the proportional hazards assumption was violated. In cases where no variables at all are identified as *stable*, no discriminatory power can be computed (denoted as –). *C*-index boosting without stability selection (without *π*_thr_) was fitted on all *p* predictors with a fixed large *m*_stop_; in case of the Cox lasso the shrinkage parameter was optimized via 10-fold cross-validation

Putting the resulting discriminatory power in Table 2 into relation with the bounds of the PFER provided in Table 1, in our simulation settings with *p*_inf_=4 the best results were achieved with a PFER (expected number of false positives) of 1 to 4. For *p*_inf_=12 and *p*_inf_=40 better results were achieved when the PFER reaches or exceeds the number of truly informative predictors *p*_inf_.

The final models of the competing Cox-lasso approach on average led to a slightly lower discriminatory power than the models from *C*-index boosting with stability selection, although in many scenarios the true informative predictors had been correctly identified. Similar to our approach, the Cox lasso also yielded the poorest results for the simulation setting with *p*_inf_=40 ($\hat {C}_{\text {Uno}} = 0.5782$) where it was clearly outperformed even by *C*-index boosting without stability selection.

### Breast cancer data

We analysed the performance of our approach also on data to build a gene signature for the prediction of the development of distant metastases in breast cancer patients. The data set (*n*=196) was collected by Desmedt et al. [27] to validate a 76-gene expression signature proposed by Wang et al. [55]. In addition to the expression levels of the 76 genes, four clinical predictor variables were considered (tumor size, estrogen receptor (ER) status, tumor grade and age). Observed metastasis-free survival ranged from 125 days to 3652 days, with 79.08 % of the survival times being censored. The data set is available on GEO (http://www.ncbi.nlm.nih.gov/geo, access number GSE 7390).

To generate independent data sets for model fitting and evaluation, we constructed 100 training and test samples via stratified subsampling (stratified for censoring). On each of the training samples, we fitted *C*-index boosting and also Cox lasso models with and without stability selection (with $q = \frac {p}{2}$ and different values of *π*_thr_). The selected genes were afterwards included together with the clinical variables in prediction models that were again fitted either via *C*-index boosting or via Cox proportional hazard models.

#### Variable selection

**Fig. 1:**
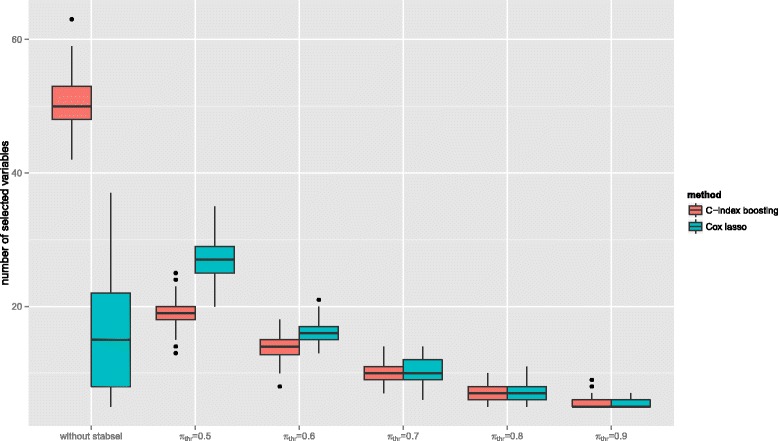
Variable selection for the breast cancer application. Number of selected variables resulting from boosting a smooth version of the *C*-index (left boxplots) and Cox lasso (right boxplots) with and without stability selection for different values of *π*_thr_. Boxplots refer to the results from 100 stratified subsamples drawn from the complete data set

Results regarding the variable selection properties of our approach and the Cox lasso are presented in Fig. 1 (*C*-index boosting left boxplots, Cox lasso right boxplots). In case of *C*-index boosting one can clearly observe how the incorporation of stability selection led to much sparser models. While *C*-index boosting on average led to models containing 50 predictors (median; range =42−63), incorporating stability selection with a minimal threshold value of *π*_thr_=0.5 yielded only 19 selected variables (median; range =13−25). Sparsity can be further enhanced by increasing the threshold: the median number of selected variables ranged from 14 variables for *π*_thr_=0.6 to 5 variables for *π*_thr_=0.9. In case of the Cox lasso, the situation was different, as already the original tuning via cross-validation yielded rather sparse models containing only 15 variables (median; range =5−37). Incorporating stability selection with low threshold values can here even identify more stable predictors than the lasso alone (e.g., 27 for *π*_thr_=0.5); only for larger threshold values the models got sparser again (e.g., 7 for *π*_thr_=0.8).

#### Discriminatory power

**Fig. 2:**
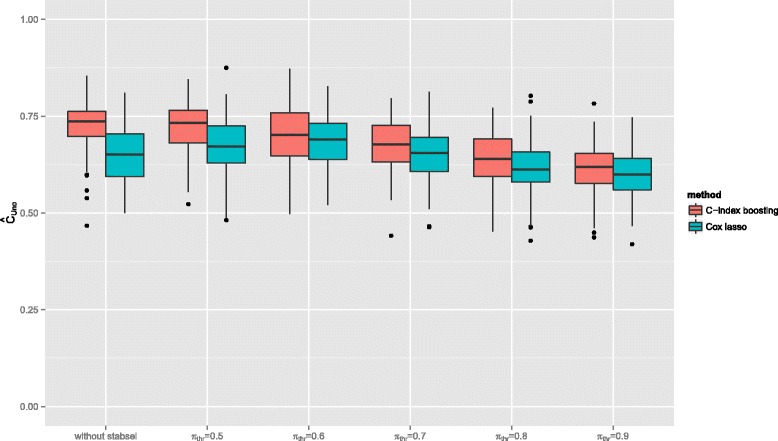
Discriminatory power for the breast cancer application. Resulting *C*-index on 100 test samples from the breast cancer application comparing both *C*-index boosting (left boxplots) and Cox lasso (right boxplots) with and without stability selection for different values of *π*_thr_

The discriminatory power of the final models (estimated via the original $\hat {C}_{\text {Uno}}$ on the test samples) is presented in Fig. 2. As expected, *C*-index boosting led to a higher discriminatory power (median $\hat {C}_{\text {Uno}} = 0.736$) than the Cox lasso (median $\hat {C}_{\text {Uno}} = 0.652$). In case of *C*-index boosting, additionally incorporating stability selection did not decrease the performance on test data ($\hat {C}_{\text {Uno}} = 0.735$ for *π*_thr_=0.5) when only a minimal threshold value was applied. Further enhancing the sparsitiy (increasing *π*_thr_), however, inevitably led to a lower discriminatory power, reflecting the trade-off between small and interpretable models and high prediction accuracy [56]. In case of the Cox lasso the situation was similar, only that again the tuning of the initial model already led to a sparser model with slightly poorer discriminatory power than the models from stability selection with low threshold value ($\hat {C}_{\text {Uno}} = 0.697$ for *π*_thr_=0.5). Generally, for any given value of *π*_thr_, the resulting $\hat {C}_{\text {Uno}}$ was higher for the boosting approach than for the Cox lasso models.

## Discussion

The numerical results from the simulation study and the breast cancer data suggest that *C*-index boosting in combination with stability selection is able to correctly identify small numbers of influential predictors in potentially high-dimensional settings.

Regarding discriminatory power, *C*-index boosting outperformed common Cox-based penalization approaches both in the simulations and in the breast cancer application. This finding is not surprising, as our approach – in contrast to Cox regression – is specifically tailored to optimize the ability of the model to differentiate between observations with smaller and larger survival times.

On the other hand, we emphasize that our approach is particular favorable for identifying sparse models, the additional sparsity resulting from stability selection does not necessarily lead to more accurate predictions. While in the simulation study, where the algorithm was confronted in most scenarios with very few informative variables and a much larger set of completely non-informative ones, the additional stability selection also led to a higher discriminatory power than standard *C*-index boosting, this result was not confirmed in the breast data application: It can be assumed that most of the 76 pre-selected genes will at least have a minor effect on the survival outcome [55]. Incorporating stability selection in this setting led to sparser models (Fig. 1), but with higher threshold values *π*_thr_ the discriminatory power decreased (Fig. 2). In fact, also the results of our simulation study have shown that for larger true models stability selection with a very strict level of error control seems to discard predictor variables that have small but non-negligible contributions to prediction accuracy. In these cases, a higher discriminatory power was achieved without the incorporation of stability selection. One could hence argue, that increasing interpretability via sparsity and getting the highest possible discriminatory power are two different goals that may not always be achievable at the same time (cf., Hothorn [56]).

In addition to the *C*-index considered in this work (see Chen et al. [57] for a similar algorithm without stability selection), various other approaches to evaluate the prediction accuracy of a survival model exist. For example, a well-established approach is to evaluate measures that emulate the *R*^2^ coefficient of explained variation by relating the likelihood of the prediction model to the respective likelihood of a null model that does not include the marker *η* [58, 59]. In contrast to the *C*-index, these measures are likelihood-based (or, in case of the Cox model, based on the partial likelihood) and are therefore dependent on the correct specification of the survival model under consideration. Another popular approach is to consider scoring rules for survival data [51, 60], which measure prediction error by the distance between the predicted and the observed survival functions of the observations in a sample. An often-used scoring rule is the Brier score, which evaluates the squared distance between survival functions [51]. Because scoring rules are based on probability estimates of the individual-specific survival functions, whereas the *C*-index is solely based on the rankings of the survival times and the marker values, the two approaches share properties that are similar to the calibration and discrimination approaches, respectively, considered in binary classification (e.g., [61]).

## Conclusion

The methodology proposed in this paper addresses the problem of variable selection in *C*-index boosting. By combining gradient boosting with stability selection, we constructed a subsampling-based estimation procedure that incorporates only the most “stable” predictor variables while controlling the per-family error rate. This property is of considerable interest in biomedical research, as the identification of a small subset of important (here, stable) markers is often considered to be a key issue in prediction modeling. As pointed out by many authors (e.g., [22]), sparse prediction models containing only a moderate number of covariates are desirable in practice for reasons of interpretability. Furthermore, measuring biomarkers is often costly, so that the implementation of a prediction model in clinical practice crucially depends on the level of sparsity of the model.

The combination of gradient boosting and stability selection may also be considered appealing from an inferential point of view. Because statistical inference in boosting models is challenging due to the partly unknown convergence properties of the algorithm and the various regularization schemes involved, very few approaches to derive covariate-wise hypothesis tests and p-values exist [62, 63]. Via stability selection, one can also compute the per-comparison error rate [64] which can be interpreted as a standard overall p-value with multiplicity correction (for details see [26]). Therefore, by controlling the number of falsely selected predictor variables, stability selection provides an alternative to covariate-wise tests for assessing the relevance of predictor variables via inferential procedures.

## Abbreviations

AUC, area under the receiver operating characteristics curve; *C*-index, concordance index; PFER, per-family error rate; FWER, family-wise error rate; ROC, receiver-operating characteristics curve

## Additional file

Additional file 1 Supporting Information. The document provides a more detailed description of the presented approach and its implementation. Furthermore, it includes a worked-out example on how *C*-index boosting with stability selection can be applied in practice. (PDF 216 kb)

Additional file 2 R Code. This R-file provides the underlying functions to reproduce the results of the simulation and the breast cancer analysis. (R 21 kb)
