# Supplementary material for: Boosting the discriminatory power of sparse survival models via optimization of the concordance index and stability selection
Source: BMC Bioinformatics. 2016 Jul 22;17:288. doi: 10.1186/s12859-016-1149-8 (PMC4957316; doi:10.1186/s12859-016-1149-8)
Supplement: Additional file 1 — Supporting Information. The document provides a more detailed description of the presented approach and its implementation. Furthermore, it includes a worked-out example on how C-index boosting with stability selection can be applied in practice. (PDF 216 kb) [file 12859_2016_1149_MOESM1_ESM.pdf]

Supporting information for
"Boosting the discriminatory power of survival models via optimization of the concordance index and stability selection"

Andreas Mayr[1], Benjamin Hofner[1], Matthias Schmid[2]

[1] Institut für Medizininformatik, Biometrie und Epidemiologie,
Friedrich-Alexander-Universität Erlangen-Nürnberg, Germany
[2] Institut für medizinische Biometrie, Informatik und Epidemiologie,
Rheinische Friedrich-Wilhelms-Universität Bonn, Germany

The complete algorithm for boosting the C-index

The aim of the algorithm is to optimize a prediction model η with respect to the concordance index via the estimator proposed by Uno et al. [1].

$$\widehat{C}_{\mathrm{Uno}}(T,\eta) \;:=\; \frac{\sum_{j,i} \frac{\Delta_j}{\hat{G}(\tilde{T}_j)^2}\,\mathrm{I}(\tilde{T}_j < \tilde{T}_i)\mathrm{I}\left(\hat{\eta}_j > \hat{\eta}_i\right)}{\sum_{j,i} \frac{\Delta_j}{\hat{G}(\tilde{T}_j)^2}\,\mathrm{I}(\tilde{T}_j < \tilde{T}_i)} \tag{1}$$

Directly using $-\widehat{C}_{\mathrm{Uno}}(T,\eta)$ as loss function for gradient boosting, however, is unfeasible because $\widehat{C}_{\mathrm{Uno}}(T,\eta)$ is not differentiable with respect to η_i.

We therefore follow the approach of Ma and Huang [2] and approximate the indicator function in by the sigmoid function $K(u) = 1/(1+\exp(-u/\sigma))$. Replacing the indicator function in (1) by its smoothed version results in the smoothed estimator

$$\widehat{C}_{\mathrm{smooth}}(T,\eta) \;=\sum_{i,k} w_{ik}\cdot\frac{1}{1\;+\;\exp\left(\frac{\hat{\eta}_k-\hat{\eta}_i}{\sigma}\right)} \tag{2}$$

with weights

$$w_{ik} \;:=\; \frac{\Delta_i\,(\hat{G}_n^L(\tilde{T}_i))^{-2}\,\mathrm{I}\left(\tilde{T}_i < \tilde{T}_k\right)}{\sum_{i,k}\Delta_i\,(\hat{G}_n^L(\tilde{T}_i))^{-2}\,\mathrm{I}\left(\tilde{T}_i < \tilde{T}_k\right)}\;. \tag{3}$$

Now, the smoothed estimator $\widehat{C}_{\mathrm{smooth}}(T,\eta)$ is differentiable with respect to the predictor η_i. Its derivative is given by

$$\frac{\partial\,\widehat{C}_{\mathrm{smooth}}(T,\eta)}{\partial\,\eta_i} \;=\sum_{k} w_{ik}\,\frac{\exp\left(\frac{\hat{\eta}_k-\hat{\eta}_i}{\sigma}\right)}{\sigma\left(1\;+\;\exp\left(\frac{\hat{\eta}_k-\hat{\eta}_i}{\sigma}\right)\right)^2}\;. \tag{4}$$

In order to use $-\widehat{C}_{\mathrm{smooth}}(T,\eta)$ as loss function to be minimized, the algrotihm needs to fit the base-learners to the negative gradient of $-\widehat{C}_{\mathrm{smooth}}(T,\eta)$ which is therefore the derivative in (4).

The complete component-wise gradient boosting algorithm for the optimization of the smoothed C-index is given as follows (for details see [3]):

---

(1) **Initialize** the estimate of the marker combination $\hat{\eta}^{[0]}$ with offset values. For example, set $\hat{\eta}^{[0]} = \mathbf{0}$, leading to $\hat{\beta}_l^{[0]} = 0$ for all components $l = 1, \ldots, p$. Choose a sufficiently large maximum number of iterations m_{stop} and set the iteration counter m to 1.

(2) **Compute** the negative gradient vector by using formula (4) and evaluate it at the marker combination $\hat{\eta}^{[m-1]}$ of the previous iteration:

$$U^{[m]} = \left(U_i^{[m]}\right)_{i=1,\ldots,n} := \left(\frac{\partial\, \widehat{C}_{\text{smooth}}(T, \hat{\eta}^{[m-1]})}{\partial\, \eta_i}\right)_{i=1,\ldots,n} .$$

(3) **Fit** the negative gradient vector $U^{[m]}$ separately to each of the components of X via the base-learners $b_l(\cdot)$:

$$U^{[m]} \xrightarrow{\text{fitted by}} \hat{b}_l^{[m]}(x_l) \quad \text{for } l = 1, ..., p.$$

(4) **Select** the component l^* that best fits the negative gradient vector according to the least squares criterion, i.e., select the base-learner b_{l^*} defined by

$$l^* = \underset{1 \le l \le p}{\operatorname{argmin}} \sum_{i=1}^{n} \left(U_i^{[m]} - \hat{b}_l^{[m]}(x_l)\right)^2 .$$

(5) **Update** the marker combination $\hat{\eta}$ for this component:

$$\hat{\eta}^{[m]} \leftarrow \hat{\eta}^{[m-1]} + \text{sl} \cdot \hat{b}_{l^*}^{[m]}(x_{l^*}) ,$$

where sl is a small step length ($0 < \text{sl} \ll 1$). A common choice for this value is $\text{sl} = 0.1$; as a result only 10% of the fit of the base-learner is added to the current model [4,5].

As only the base learner $\hat{b}_{l^*}$ was selected, only the effect of component l^* is updated ($\hat{\beta}_{l^*}^{[m]} = \hat{\beta}_{l^*}^{[m-1]} + \text{sl} \cdot \hat{b}_{l^*}^{[m]}(x_{l^*})$) while all other effects stay constant ($\hat{\beta}_l^{[m]} = \hat{\beta}_l^{[m-1]}$ for $l \neq l^*$).

(6) **Stop** if $m = m_{\text{stop}}$. Else increase m by one and go back to step (2).

Implementation

The most flexible implementation of gradient boosting for statistical modelling, which is also relatively easy to extend [6], is the `mboost` [7] add-on package for the Open Source programming environment `R` [8]. For a tutorial on the how to apply the package for practical data analysis, see [4].

For stability selection we apply the `stabsel()` function from the `stabs` [9] package, which is also incorporated in `mboost` for boosting models. It provides an implementation of the classical approach proposed by Meinshausen and Bühlmann [10] and the extended sampling scheme by Shah and Samworth [11]. For evaluating the discriminatory power of the resulting model on test data, we use the `UnoC()` function of

the `survAUC` [12] package.

To apply gradient boosting to fit linear statistical models that are optimal for the C-index in the version of Uno et al. [1], one needs to define the following `Cindex()` family to be used withon the `glmboost()` function, for details see Mayr and Schmid [3].

```
Cindex <- function (sigma = 0.1, ipcw = 1) {

  approxGrad <- function(x) {                     ## sigmoid function for gradient
    exp(x/sigma) / (sigma * (1 + exp(x/sigma))^2)
  }
  approxLoss <- function(x) {                     ## sigmoid function for loss
    1 / (1 + exp(x / sigma))
  }

  compute_weights <- function(y, w = 1, ipcw){    ## compute weights
    if(all(ipcw == 1)){
      ipcw_wow <- IPCweights(y[w != 0,])
      ipcw <- numeric(nrow(y))
      ipcw[w!=0] <- ipcw_wow
    }
    survtime <- y[,1]
    n <- nrow(y)
    wweights <- matrix( (ipcw)^2, nrow = n, ncol = n)
    weightsj <- matrix(survtime, nrow = n, ncol = n)
    weightsk <- matrix(survtime, nrow = n, ncol = n, byrow = TRUE)
    weightsI <- ifelse(weightsj == weightsk, .5,
                       (weightsj < weightsk) + 0) - diag(.5, n,n)
    wweights <- wweights * weightsI
    Wmat <- w %o% w
    wweights <- wweights * Wmat
    wweights <- wweights / sum(wweights)
    rm(weightsI); rm(weightsk); rm(weightsj)
    return(wweights)
  }


  ngradient = function(y, f, w = 1) {              ## negative gradient
    if (!all(w %in% c(0,1)))
      stop(sQuote("weights"), " must be either 0 or 1 for family ",
           sQuote("UnoC"))
    survtime <- y[,1]
    event <- y[,2]
    if (length(w) == 1) w <- rep(1, length(event))
    if (length(f) == 1) {
      f <- rep(f, length(survtime))
    }
    n <- length(survtime)
    etaj <- matrix(f, nrow = n, ncol = n, byrow = TRUE)
```

```
    etak <- matrix(f, nrow = n, ncol = n)
    etaMat <- etak - etaj
    rm(etaj); rm(etak);
    weights_out <- compute_weights(y, w, ipcw)
    M1 <- approxGrad(etaMat) * weights_out
    ng <- colSums(M1) - rowSums(M1)
    return(ng)
  }

  risk = function(y, f, w = 1) {                  ## empirical risk
    survtime <- y[,1]
    event <- y[,2]
    if (length(f) == 1) {
      f <- rep(f, length(y))
    }
    n <- length(survtime)

    etaj <- matrix(f, nrow = n, ncol = n, byrow = TRUE)
    etak <- matrix(f, nrow = n, ncol = n)
    etaMat <- (etak - etaj)
    rm(etaj); rm(etak);
    weights_out <- compute_weights(y, w, ipcw)
    M1 <- approxLoss(etaMat) * weights_out
    return(- sum(M1))
  }

  Family(                                        ## build the family object
    ngradient = ngradient,
    risk = risk,
    weights = "zeroone",
    offset = function(y, w = 1) {0},
    check_y = function(y) {
      if (!inherits(y, "Surv"))
        stop("response is not an object of class ", sQuote("Surv"),
             " but ", sQuote("family = UnoC()"))
      y},
    rclass = function(f){},
    name = paste("Concordance Probability by Uno")
  )
}
```

Example

We will briefly demonstrate how to apply the `Cindex()` family in practice in combination with stability selection to derive the optimal combination biomarkers. We will use the van de Vijver et al. [13] data set of 144 lymph node positive breast cancer patients. The data set is publicly available as part of the `R` add-on package `penalized` [14]. The 70-gene signature for metastasis-free survival after surgery was originally developed by van't Veer et al. [15].

We first split the data set in 100 training observations and 44 test observations. Model fitting is carried out by the `glmboost()` function of the `mboost` package. As linear models are the default base-learners for `glmboost()`, no additional base-learner has to be specified. As appropriate `family` object we specify the `Cindex` family provided above.

```
## load add-on packages
library(penalized) ## for the data set
library(mboost)    ## for boosting
library(survAUC)   ## for evaluation

data(nki70)        ## loading the data
source("Cindex.R") ##  loading the family defined above

## split the data set in training and test sample (simplified):
dtrain <- nki70[1:100,]
dtest  <- nki70[101:144,]

## extract the 70 gene expression levels
expr <- as.matrix(dtrain[,8:77])

## fit a model via the glmboost() function
## formula : defines the candidate model; the response here is the survival
##           object Surv(time, event); via '~ ." all remaining variables
##           in the data set serve as possible predictors
## data    : defines the data -> training sample
## y       : alternative to 'formula', specifies the response
## x       : alternative to 'formula', specifies the predictors via a matrix
## family  : defines the optimization problem (in this case the C-index)
##           sigma is the smoothing parameter of the sigmoid function that
##           approximates the indicator functions. The default here is 0.01.
## control : defines other boosting-specific tuning parameters like the
##           stopping iteration mstop or the step-length nu; trace = TRUE is
##           only for convenience (shows the trace of the empirical risk).


## compute the IPC weights (optional)
## avoids re-computing in each iteration and on each subsample
ipcw <- IPCweights(Surv(dtrain$time, dtrain$event))


## fit an initial model only with gene expression levels:
mod1 <- glmboost(y = Surv(dtrain$time, dtrain$event), x = expr,
                 family = Cindex(sigma = 0.01, ipcw = ipcw ),
                 control = boost_control(mstop = 100, trace = TRUE, nu = 0.1))

## The stopping iteration can be changed afterwards:
mstop(mod1) <- 500
```

```
## Now take a look at the selected genes
coef(mod1)

## stability selection via stabsel() for a boosting model
## object        : the initial boosting model fitted by mboost
## q             : number of selected base-learners (predictors) on each subsample
## cutoff        : threshold value (pi_thr) for the minimum selection probability
##                 necessary to be included in the final model
## PFER          : upper bound for the per-family error rate; is fixed if q and
##                 cutoff are specified, can be computed via the function
##                 stabsel_parameters() for information.
## sampling.type : specify 'SS' for the version of Shah & Samworth (2013)
##                 with complementarty pairs, 'MB' or the original sampling
##                 scheme of Meinshausen & Buehlmann (2010)
## assumption    : only applies for sampling.type = 'SS'. Can be 'unimodal'
##                 or 'r-concave'; for details see Shah & Samworth (2013) and
##                 Hofner et al. (2015), we used 'r-concave' throughout the
##                 analysis for this paper.

## carry out stability selection
## LONG RUNTIME (can take around 15 minutes on standard machine)
## automatically incorporates parallel computing if mclapply is available
set.seed(123)
stab <- stabsel(mod1, q = 30, cutoff = 0.7, sampling.type = "SS",
                assumption = "r-concave")

## check the selected genes
stab$selected

## check the PFER
stab$PFER

## compute PFER for other parameters
stabsel_parameters(p = 70, q = 30, cutoff = 0.8,  sampling.type = "SS",
                   assumption = "r-concave" )

## build final models with those genes and clinical predictors
form <- as.formula(paste("Surv(time, event) ~ Diam + N + ER + Grade + Age +",
                         paste(names(stab$selected), collapse = "+")))

## fit final model on training data
mod1 <- glmboost(form, family = Cindex(sigma = 0.01, ipcw = ipcw),
                 control = boost_control(mstop = 1000, trace = TRUE, nu = 0.1),
                 data = dtrain)

## compute the predictions on test data
preds <-  predict(mod1, newdata = dtest)

## evaluate the discriminatory power by Uno's estimator
```

```
UnoC(Surv(dtrain$time, dtrain$event),  Surv(dtest$time, dtest$event), lpnew = -preds)

## use different values for the cutoff without re-computing: e.g., cutoff = 0.5
form05 <- as.formula(paste("Surv(time, event) ~ Diam + N + ER + Grade + Age +",
                           paste(names(stab$max)[stab$max >0.5], collapse = "+")))

## re-fit model
mod2 <- glmboost(form05, family = Cindex(sigma = 0.01, ipcw = ipcw),
                 control = boost_control(mstop = 1000, trace = TRUE, nu = 0.1),
                 data = dtrain)

## evaluate again
UnoC(Surv(dtrain$time, dtrain$event), Surv(dtest$time, dtest$event),
     lpnew = -predict(mod2, newdata = dtest))
```

References

1. Uno H, Cai T, Pencina MJ, D'Agostino RB, Wei LJ. On the C-statistics for Evaluating Overall Adequacy of Risk Prediction Procedures with Censored Survival Data. Statistics in Medicine. 2011;30(10):1105–1117.

2. Ma S, Huang J. Regularized ROC Method for Disease Classification and Biomarker Selection with Microarray Data. Bioinformatics. 2005;21(24):4356–4362.

3. Mayr A, Schmid M. Boosting the Concordance Index for Survival Data – A Unified Framework to Derive and Evaluate Biomarker Combinations. PloS ONE. 2014;9(1):e84483.

4. Hofner B, Mayr A, Robinzonov N, Schmid M. Model-Based Boosting in R: A Hands-on Tutorial Using the R Package mboost. Computational Statistics. 2014;29:3–35.

5. Mayr A, Binder H, Gefeller O, Schmid M. The Evolution of Boosting Algorithms - From Machine Learning to Statistical Modelling. Methods of Information in Medicine. 2014;53(6):419–427.

6. Mayr A, Binder H, Gefeller O, Schmid M. Extending Statistical Boosting - An Overview of Recent Methodological Developments. Methods of Information in Medicine. 2014;53(6):428–435.

7. Hothorn T, Bühlmann P, Kneib T, Schmid M, Hofner B. mboost: Model-Based Boosting; 2015. R package version 2.5-0. Available from: `http://CRAN.R-project.org/package=mboost`.

8. R Development Core Team. R: A Language and Environment for Statistical Computing. Vienna, Austria; 2015. ISBN 3-900051-07-0. Available from: `http://www.R-project.org`.

9. Hofner B, Hothorn T. stabs: Stability Selection with Error Control; 2015. R package version 0.5-1. Available from: `http://CRAN.R-project.org/package=stabs`.

10. Meinshausen N, Bühlmann P. Stability Selection (with Discussion). Journal of the Royal Statistical Society Series B. 2010;72:417–473.

11. Shah RD, Samworth RJ. Variable Selection with Error Control: Another Look at Stability Selection. Journal of the Royal Statistical Society: Series B (Statistical Methodology). 2013;75(1):55–80.

12. Potapov S, Adler W, Schmid M. survAUC: Estimators of prediction accuracy for time-to-event data.; 2012. R package version 1.0-5. Available from: `http://CRAN.R-project.org/package=survAUC`.

13. van de Vijver MJ, He YD, van't Veer LJ, Dai H, Hart AAM, Voskuil DW, et al. A Gene-Expression Signature as a Predictor of Survival in Breast Cancer. New England Journal of Medicine. 2002;347(25):1999–2009.

14. Goeman JJ. L_1 Penalized Estimation in the Cox Proportional Hazards Model. Biometrical Journal. 2010;551(1):70–84.

15. van't Veer LJ, Dai HY, van de Vijver MJ, He YDD, Hart AAM, Mao M, et al. Gene Expression Profiling Predicts Clinical Outcome of Breast Cancer. Nature. 2002;415(6871):530–536.
